# Intrasession repeatability and agreement of a new method to measure the foveal fixation axis

**DOI:** 10.7717/peerj.14942

**Published:** 2023-02-24

**Authors:** Oscar Garcia Espinilla, Irene Sanchez, Raul Martin

**Affiliations:** 1 Optometry Research Group, IOBA Eye Institute, School of Optometry, Universidad de Valladolid, Valladolid, Castilla y Leon, Spain; 2 Instituto Universitario de Oftalmobiología Aplicada (IOBA), Universidad de Valladolid, Valladolid, Castilla y Leon, Spain; 3 Departamento de Física Teórica, Atómica y Óptica, Universidad de Valladolid, Valladolid, Castilla y Leon, Spain

**Keywords:** Nasopupillary distance, Foveal fixation axis, Ophthalmic lens prescription, Intrasession repeatability, Agreement

## Abstract

**Purpose:**

Ophthalmic lens adaptation, particularly with progressive addition lenses, requires accurate measurements of the patient nasopupillary distance (NPD) and interpupillary distance (IPD), which are usually collected using the pupil centre as a reference. However, differences between the pupil centre and visual or foveal axis could induce some subsidiary effects of correcting lenses. This study aimed to assess the intrasession repeatability of a new prototype (Ergofocus®; Lentitech, Barakaldo, Spain) that can measure the foveal fixation axis (FFA) distance and assess the agreement with the NPD measurements collected using a traditional method (frame ruler).

**Methods:**

The FFA at far and near distances was measured three consecutive times in 39 healthy volunteers to determine the intrasession repeatability according to the British Standards Institute and International Organization for Standardization. Additionally, the FFA and NPD (standard frame ruler) were measured in 71 healthy volunteers and compared using Bland–Altman analysis. Two blinded experienced practitioners conducted each FFA and NPD measurement.

**Results:**

The FFA measurements showed acceptable repeatability at far distances (right eye (RE): Sw = 1.16 ± 0.76 mm and coefficient of variation (CV) = 3.92 ± 2.51%; left eye (LE) Sw = 1.11 ± 0.79 mm and CV = 3.76 ± 2.51%) and at near distances (RE: Sw = 0.97 ± 0.85 mm and CV = 3.52 ± 3.02%; LE: Sw = 1.17 ± 0.96 mm and CV = 4.54 ± 3.72%). Additionally, agreement with the NPD showed large differences at far distances (RE: −2.15 ± 2.34, LoA = −6.73 to 2.43 mm (*P* < 0.001); LE: −0.61 ± 2.62, LoA = −5.75 to 4.53 mm (*P* = 0.052)) and near distances (RE: −3.08 ± 2.80, LoA −8.57 to 2.42 mm (*P* < 0.001); LE: −2.97 ± 3.97, LoA: −10.75 to 4.80 mm (*P* < 0.001)).

**Conclusions:**

FFA measurements showed clinically acceptable repeatability at both far and near distances. Agreement with the NPD measured using a standard frame ruler showed significant differences, suggesting that both measurements are not interchangeable in clinical practice to prescribe and center ophthalmic lenses. Further research is necessary to assess the impact of FFA measurement in ophthalmic lens prescriptions.

## Introduction

Refractive errors (myopia, hyperopia and astigmatism) affect most of the worldwide population (more than 2.3 billion people in the world) (Naidoo & Jaggernath, 2012), and presbyopia (physiological age-related loss of lens ability to near focus after 45 years of age) currently affects approximately one billion people globally (Wong et al., 2008) and is expected to increase significantly by 2050 (United Nations, 2019) because of societal ageing. Refractive errors must be corrected to restore the correct visual function using spectacles, contact lenses (Fernandes et al., 2013) or refractive surgery (Van Cauwenberge & Rakic, 2014), but ophthalmic lenses, particularly progressive addition lenses (PALs) in presbyopia, are the popular option for most users (Charman, 2014).

Ophthalmic lens prescription, particularly in high refractive errors or PAL, requires accurate measurements of some facial parameters of the patient (Han, Graham & Lin, 2011; McMahon, Irving & Lee, 2012) that are measured using different methods. Manual measurement (Walsh & Pearce, 2009) using a traditional frame ruler is one of the most popular (whose precision is commonly limited to 1 mm, which is its minimum unit of measurement), but it is not free of errors such as parallax error and examiner experience (Pointer, 2012). Other devices such as pupillary gauges use the pupil centre as a reference (Walsh & Pearce, 2009) to align it with the prescribed lens optical center (Anderson, 1954) are usually used.

However, using the pupil centre as a lens centering reference implies a misconception because the pupil centre usually does not meet the visual axis. Although no consistent definition is available in the literature (Chang & Waring, 2014), the visual axis should be the line connecting the fixation point with the foveola passing through the two nodal points of the eye (Rabbetts, 2007) that are simplified as a single point in some reports (Chang & Waring, 2014). The angle between the visual axis and pupillary axis (line from the centre of the entrance pupil that perpendicularly passes through the centre of curvature of the cornea) conforms to the angle kappa (Chang & Waring, 2014; Rabbetts, 2007), which is usually clinically identified as the distance between the corneal light reflex and pupil centre (Park, Oh & Chuck, 2012). Therefore, current methods for ophthalmic lenses centering using pupil centres to compensate for refractive errors present limitations that could affect user vision and spectacle comfort of wear.

PALs are one-piece spectacle design lenses with a progression of plus power across the lens surface from the distance prescription (the upper part of the lens) to the near prescription (the lower part of the lens, which is usually nasally decentred). Change in PALs power surface, induces lateral aberrations, as explained by the Minkwitz theorem (Sheedy et al., 2005). Consequently, small errors in PAL centration could induce several distortions (aberrations) in the users visual field with a high impact on visual performance and user’s comfort with PAL. These aberrations cause PAL users an adaptation process (Boroyan et al., 1995) between 1 and 3 weeks (Han, Graham & Lin, 2011; Jaschinski et al., 2015) when PAL is correctly prescribed and fitted. To minimize the impact of Minkwitz astigmatism on users’ vision (Chamorro et al., 2018), free-form customized PALs have been developed, but these lenses require the personalization of the lens design to the users’ facial parameters (Chamorro et al., 2018).

A new prototype (Ergofocus®; Lentitech, Barakaldo, Spain) was developed to measure the foveal fixation axis distance (FFA) to improve ophthalmic lens prescription. The FFA is defined as the imaginary line that directly links the fixation point and fovea (Chang & Waring, 2014), and its measurement could allow the centering of the lens optical centre with the point through the eye conduct the fixation (where eye is truly looking through), considering the difference between foveal axis and pupillary center (angle kappa) and avoiding to center ophthalmic lens in an approximate point like the pupillary center. Better or precise lens centration could improve satisfaction with optical compensation of refractive error, particularly in high prescriptions or in PALs, which are more likely to produce discomfort and sometimes drop out of lens use.

Consequently, this study aimed to describe the intrasession repeatability (Garcia-Espinilla et al., 2022) of FFA distance measurement and assess the agreement (Giavarina, 2015) with the pupillary distance measured using a standard frame ruler to assess the possible use of the FFA in clinical practice to prescribe and fit ophthalmic lenses.

## Methods

### Subjects

This study involved 71 healthy subjects between the ages of 44 and 64 years with a visual acuity equal to or better than 20/30 to allow accurate fixation at far and near distances. Patients with severe systemic disease (multiple sclerosis, Parkinson’s disease, Alzheimer’s disease, cancer, and others), advanced glaucoma or visual acuity under 20/30 were excluded from the study. Written informed consent was obtained from each subject after the Human Sciences Ethics Committee of Valladolid Area-Este Clinic Hospital (Castilla y Leon Public Health System-SACYL) approved the study (PI 19-1194). All the subjects were treated in accordance with the Declaration of Helsinki.

The nasopupillary distance and interpupillary distance were measured using a traditional frame ruler, and the FFA distance was also measured using the new Ergofocus device in all 71 subjects to conduct the agreement analysis. Both measurements were conducted in the same session by two blinded experienced practitioners. However, in only 39 patients, the FFA distance was measured three consecutive times to conduct the repeatability assessment.

### Measurement procedure

**Figure 1:**
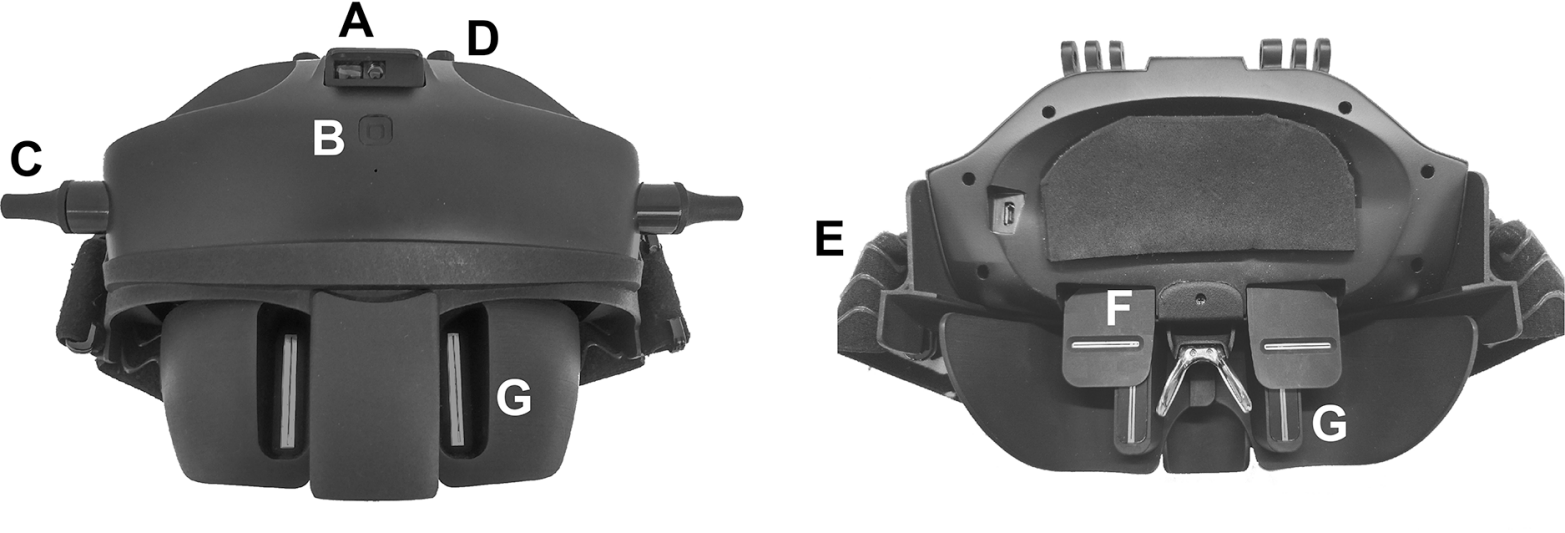
Front (left) and back (right) image of the Ergofocus® device designed for FFA distance measurement. (A) Distance sensor. (B) On/Off button. (C) Vertical slits displacement control. (D) Horizontal slits displacement control. (E) Rubber band. (F) Horizontal slit. (G) Vertical slit.

A device designed to measure the FFA distance (Ergofocus^®^; Lentitech Inc, Barakaldo, Spain) (Ergofocus, 2022) (Fig. 1) and a traditional frame ruler were used to conduct the measurements. The Ergofocus device comprises two moveable slits located in front of each eye (one horizontal and one vertical). The FFA distance is determined as the distance from the vertical slit of each eye until the middle of the device when patients see the target through both vertical and horizontal slits and is automatically saved in the device. The device has a laser sensor (top and middle located) to measure the target distance (at far and near distances) at which the FFA measurement is made. After each measurement, a patient file with all the measurement data (repeated measurements and far and near distance) is generated and saved in a tablet app *via* Bluetooth.

The patient wore the device on their head, fixed in place with rubber bands and resting on their nose. To measure the FFA distance in far vision, a fixation object was placed at 6 m. To measure the right eye FFA distance, the left eye was occluded, and right eye slits were manually moved by the examiner until the patient could see the fixation object centred in their visual field and vice versa for the left eye. When left eye measurement was completed, the examiner checked that the fixation point was centred binocularly. To measure the FFA distance in near vision, the process was the same as that for far distance except that the fixation point was placed at comfortable reading distance for the patient (measured by the device sensor).

Frame ruler measurements were conducted following a standard procedure (Walsh & Pearce, 2009; Garcia-Espinilla et al., 2022). To collect far vision pupillary distance measurements, the patient (wearing the frame) and examiner were seated facing each other with an approximate distance of 1 m. First, the examiner closed his/her right eye, the patient looked at the examiner’s left pupil with the right eye, and then the examiner marked the patient’s right eye pupil centre with a marker on the glass of the frame. This procedure was repeated with the examiner closing his/her left eye and the patient looking at the examiner’s right eye with the left eye. Next, the patient removed the frame, and using the traditional frame ruler, the examiner measured the distance between the two marks (interpupillary distance) and distance from the centre of the bridge (nasopupillary distance). This modification of Viktorin’s method (Walsh & Pearce, 2009) guarantees the correct measurement of the nasopupillary distance when the frame is not symmetrically centred—for example, because of nose asymmetry. To collect near vision measurements, the examiner was placed at the reading distance of the patient (this distance was measured using a tailor’s tape), and the patient looked at the examiner’s nose. Next, the examiner uses a marker to mark the centre of the patient’s pupils and, with the frame ruler, measures the distance between both marks (interpupillary distance) and with the centre of the frame bridge (nasopupillary distance).

### Statistical analysis

Statistical analysis was performed using SPSS for Windows software (version 23.0; Chicago, IL, USA). The nonparametric data distribution of variables was verified using the Kolmogorov–Smirnov test (*P* < 0.05 indicated that the data were not normally distributed). Data for the collected variables were presented as means, standard deviations (SDs) and ranges.

For intrasession repeatability, the set of three consecutive measurements obtained in the same session of each parameter was calculated following the definitions of repeatability according to the British Standards Institute and International Organization for Standardization (British Standards Institute (BSI) and International Organization for Standardization (ISO), 1994): within-subject standard deviation (Bland, 2000), repeatability (Bland, 2000) (2.77× within-subject standard deviation, which defines the difference between two measurements of the same volunteer for 95% of the pairs of observations), coefficient of variation (Bland, 2000) (percentage value of the variation of the measurement and defined as the ratio of the within-subject standard deviation (Sw) to the overall mean (coefficient of variation = within-subject standard deviation/mean × 100 (%))) and the intraclass correlation coefficient (ICC; classified as follows: less than 0.75 = poor agreement; 0.75 to <0.90 = moderate agreement; ≥0.90 = high agreement; McGraw & Wong, 1996). The differences between pairs of repeated measurements were plotted against the mean of both measurements in each eye at far and near distances. The limit of agreement (LoA) (mean ± 1.96 standard deviations) (Giavarina, 2015; Bland, 2000; Carkeet, 2015) and exact 95% confidence interval for the repeatability of the LoA (Carkeet, 2015) were calculated.

Agreement analysis was conducted following Bland–Altman recommendations. Differences between the measurements of two different devices were presented *vs* the mean of these two measurements. The ninety-five percent LoA was calculated (mean difference ±1.96 × SD of the mean difference) (Giavarina, 2015; Bland, 2000; Carkeet, 2015). Linear regression analysis was used to assess the effect of the overall magnitude of the mean distance on the differences between the measurements of both devices, and the R^2^ correlation coefficient was calculated (*P* < 0.05 was considered statistically significant). Exact 95% confidence intervals for the repeatability of the LoA were also calculated (Carkeet, 2015).

Comparisons between devices for each measured parameter were made using paired *t* test or Wilcoxon nonparametric paired tests depending on the sample distribution (*P* < 0.05 was considered statistically significant).

## Results

Seventy-one healthy subjects (37 women and 34 men) with an average age of 54.01 ± 4.50 years (44 to 64 years) and a spherical equivalent of −0.70 ± 2.52 D (−7.50 to +4.00 D) were enrolled in the study. Near distance was on average 33.14 ± 6.85 cm (ranged from 19.80 to 60.10 cm). All the subjects participated in the agreement study, but only 39 were also enrolled in the repeatability study (17 women and 22 men, with an average age of 53.34 ± 4.33 years (44 to 62 years), a spherical equivalent of −0.61 ± 2.52 (−7.50 to +4.00 D) and near distance of 32.66 ± 5.04 cm (19.80 to 42.00) cm).

### Repeatability analysis

**Figure 2:**
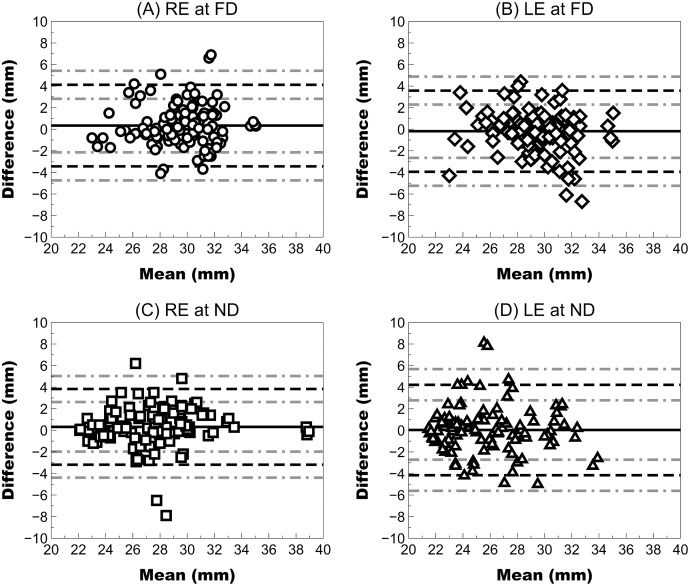
Bland–Altman plot showing the repeatability of Ergofocus FFA measurements of the right (RE) and left eye (LE) at a far distance (FD) and a near distance (ND). Mean difference (continuous black line), limit of agreement (LoA) (discontinuous black line) and 95% CI for the LoA (discontinuous grey line) were plotted as follows: (A) RE at the FD: mean difference of 0.34 ± 1.93 mm, LoA (95% CI) ranging from −3.44 [−4.74 to −2.14] to 4.12 [2.82–5.42] and correlation coefficient (R^2^) of <0.01 (*P* = 0.654); (B) LE at FD: mean difference of −0.18 ± 1.92 mm, LoA (95% CI) ranging from −3.94 [−5.24 to −2.65] to 3.58 [2.29–4.88] and R^2^ coefficient of 0.02 (*P* = 0.101); (C) RE at FD: mean difference of 0.32 ± 1.79 mm, LoA (95% CI) ranging from −3.19 [−4.39 to −1.98] to 3.83 [2.62–5.03] and R^2^ coefficient of <0.01 (*P* = 0.500); (D) LE at ND: mean difference of 0.03 ± 2.14 mm, LoA (95% CI) ranging from −4.16 [−5.61 to −2.72] to 4.22 [2.78–5.67] and R^2^ coefficient of 0.03 (*P* = 0.570).

FFA measurements showed acceptable repeatability for clinical use in both right and left eyes with a CV lower than 5%, a Sw close 1 mm and an ICC higher than 0.89 at far and near distances (Table 1 and Fig. 2).

**Table 1:** Summary of the descriptive and intrasession repeatability coefficients (Sw, CV, ICC mean difference and LoA) for FFA measurements at far and near distances (*n* = 39).

	FFA FD RE	FFA FD LE	FFA ND RE	FFA ND LE
Descriptive				
Mean value ± SD (mm)	29.54 ± 2.37	29.64 ± 2.92	27.42 ± 3.18	25.90 ± 2.99
Range (mm)	23.40 to 34.83	24.27 to 35.93	22.12 to 38.83	21.57 to 33.23
CI 95% (mm)	[28.77–30.30]	[28.70–30.59]	[26.39–28.45]	[24.93–26.87]
Intrasession repeatability coefficients				
Sw (mm)	1.16 ± 0.76	1.11 ± 0.79	0.97 ± 0.85	1.17 ± 0.96
Rep (mm)	3.21 ± 2.11	3.07 ± 2.20	2.68 ± 2.35	3.25 ± 2.66
CV (%)	3.92 ± 2.51	3.76 ± 2.51	3.52 ± 3.02	4.54 ± 3.72
ICC	0.89	0.93	0.95	0.91
Mean diff ± SD (mm)	0.34 ± 1.93	−0.18 ± 1.92	0.32 ± 1.79	0.03 ± 2.14
LoA (mm)	−3.44 to +4.12	−3.94 to +3.58	−3.19 to +3.83	−4.16 to +4.22
95% CI lower LoA (mm)	[−4.74 to −2.14]	[−5.24 to −2.65]	[−4.39 to −1.98]	[−5.61 to −2.72]
95% CI upper LoA (mm)	[+2.82 to +5.42]	[+2.29 to +4.88]	[+2.62 to +5.03]	[+2.78 to +5.67]

**Note:**

FFA, foveal fixation axis; FD, far distance; RE, right eye; LE, left eye; ND, near distance; SD, standard deviation; CI, confidence interval; Sw, within-subject standard deviation; Rep, repeatability; CV, coefficient of variation; ICC, intraclass correlation coefficient; Diff, difference; LoA, limit of agreement.

### Agreement analysis

**Figure 3:**
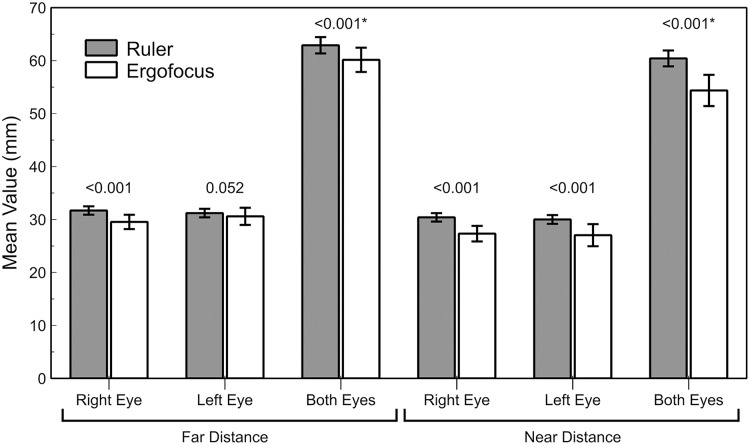
Bar graph representation of the traditional ruler and Ergofocus device measurements at far and near distances. *P* value calculated using the Wilcoxon non parametric paired test is shown (*highlighted *P* value calculated using paired t test).

The differences between traditional ruler measurements (nasopupillary distance or interpupillary distance) and Ergofocus FFA showed worse agreement (high differences with wide LoA) and statistically significant differences (*P* < 0.001) in all assessed parameters except in left eye measurements at far distances (*P* = 0.052, Z = −1.941) (Table 2 and Fig. 3).

**Table 2:** Summary of the agreement between all the distances measured using the frame ruler and Ergofocus (FFA) device

		NPD (mm)		FFA (mm)		Mean Diff ± SD(mm)	*P* Value
		Mean ± SD (Range)	IC 95%	Mean ± SD (Range)	IC 95%		
FD	RE	31.70 ± 1.68 (28.00 to 36.00)	[31.31–32.10]	29.55 ± 2.84 (22.20–36.80)	[28.88–30.23]	−2.15 ± 2.34	<0.001 (Z = −5.853)
	LE	31.21 ± 1.71 (27.00 to 36.00)	[30.81–31.62]	30.60 ± 3.41 (24.00–40.70)	[29.79–31.41]	−0.61 ± 2.62	0.052 (Z = −1.941)
	BE	62.90 ± 3.26 (55.00 to 72.00)	[62.13–63.67]	60.15 ± 4.82 (48.50–76.10)	[59.01–61.30]	−2.75 ± 2.93	<0.001 (t = 7.904, df = 70)*
ND	RE	30.41 ± 1.70 (26.00 to 35.00)	[30.01–30.81]	27.33 ± 3.10 (21.40–34.90)	[26.60–28.07]	−3.08 ± 2.80	<0.001 (Z = −6.409)
	LE	30.01 ± 1.75 (26.00 to 34.00)	[29.60–30.43]	27.04 ± 4.41 (17.60–40.80)	[26.00–28.09]	−2.97 ± 3.97	<0.001 (Z = −5.340)
	BE	60.42 ± 3.26 (52.00 to 69.00)	[59.69–61.19]	54.37 ± 6.25 (43.50–74.90)	[52.90–55.85]	−6.05 ± 5.35	<0.001 (t = 9.520, df = 70)*

**Note:**

NPD, nasopupillary distance; FFA, foveal fixation axis; FD, far distance; ND, near distance; RE, right eye; LE, left eye; BE, both eyes measured. *P* value calculated using the Wilcoxon non parametric paired test except in * values that were calculated using paired t test.

**Figure 4:**
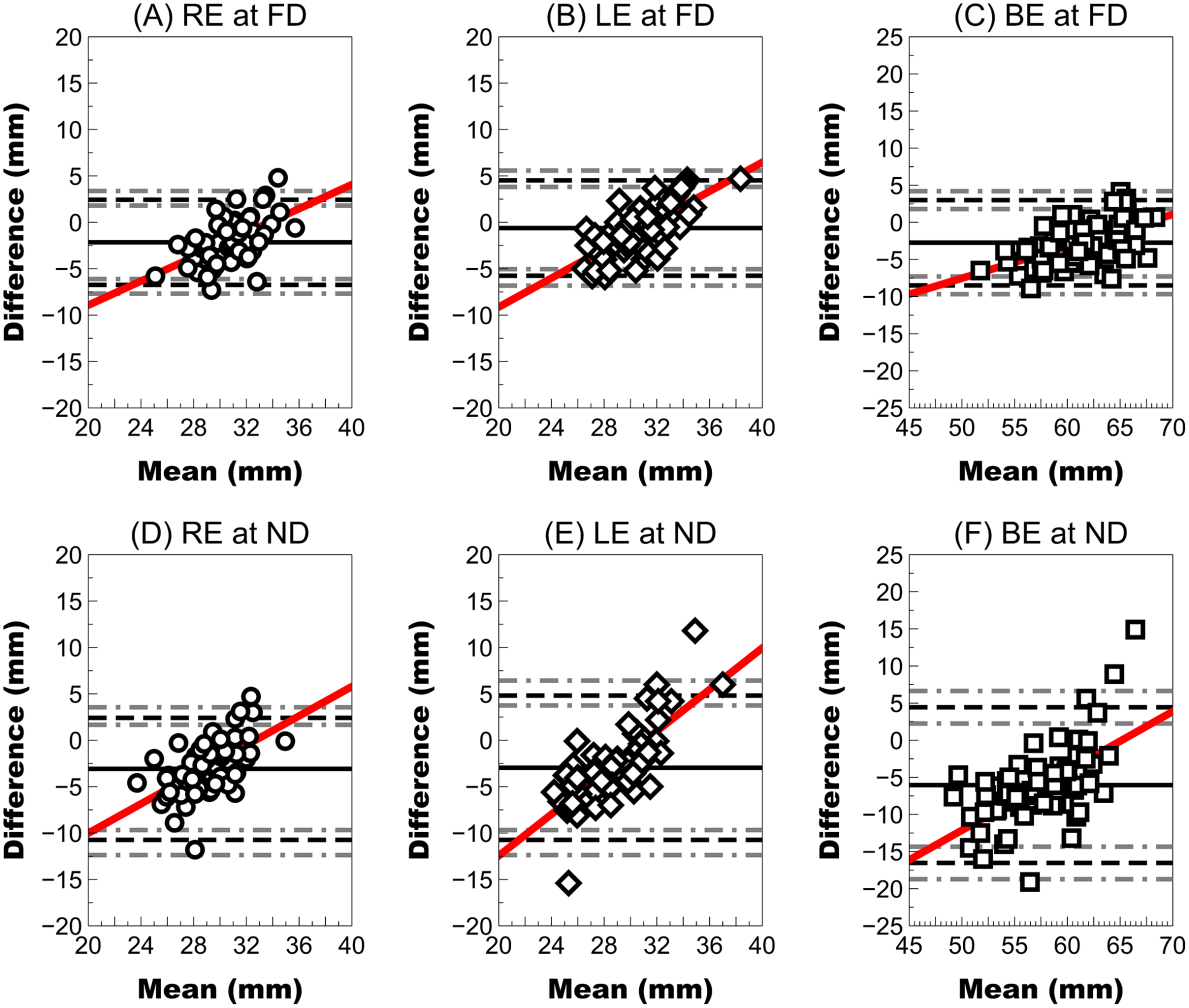
Bland–Altman plot showing the agreement between the ruler pupillary distances and Ergofocus FFA measurements of the right, left eye and both eyes at a far distance (FD) and a near distance (ND). Mean difference (continuous line), limit of agreement (LoA) (discontinuous black line) and 95% CI for the LoA (discontinuous grey line) were plotted as follows: (A) RE at FD: mean difference of −2.15 ± 2.34 mm, LoA (95% CI) ranging from −6.74 [−7.69 to −6.11] to 2.44 [1.81–3.39] and correlation coefficient (R^2^) of 0.31 (*P* < 0.001); (B) LE at FD: mean difference of −0.61 ± 2.62 mm, LoA (95% CI) ranging from −5.75 [−6.81 to −5.04] to 4.53 [3.82–5.59] and R^2^ coefficient of 0.49 (*P* < 0.001); (C) BE at FD: mean difference of −2.75 ± 2.93 mm, LoA (95% CI) ranging from −8.49 [−9.69 to −7.29] to 2.99 [1.79–4.19] and R^2^ coefficient of 0.31 (*P* < 0.001); (D) RE at ND: mean difference of −3.08 ± 2.80 mm, LoA (95% CI) ranging from −8.57 [−9.71 to −7.82] to 2.41 [1.66–3.55] and R^2^ coefficient of 0.34 (*P* < 0.001); (E) LE at ND: mean difference of −2.97 ± 3.97 mm, LoA (95% CI) ranging from −10.75 [−12.37 to −9.69] to 4.81 [3.75–6.43] and R^2^ coefficient of 0.58 (*P* < 0.001); (F) BE at ND: mean difference of −6.05 ± 5.35 mm, LoA (95% CI) ranging from −16.54 [−18.73 to −14.34] to 4.44 [2.24–6.63] and R^2^ coefficient of 0.39 (*P* < 0.001).

At far distances, the right eye showed a higher mean difference (close 2 mm with LoA larger ±4 mm) than the left eye (mean difference <1 mm), but the wide LoA ranged between approximately ±5 mm. In both eyes, the mean difference and LoA were slightly larger (mean difference close 3 mm and LoA close ±6 mm). However, agreement at near distances was worse than at far distances because both right and left eyes showed a mean difference close to 3 mm with a wider LoA (higher than ±10 mm) (Fig. 4). The interpupillary distance and FFA in both eyes showed the largest mean difference of all parameters and the widest LoA (±10 mm).

## Discussion

Correct centration of ophthalmic lenses (Alderson et al., 2016) is necessary to achieve patient satisfaction with optical correction. However, ophthalmic lenses are usually centred using the pupillary centre as a reference, and this procedure must be performed on the visual or foveal fixation axis (Chang & Waring, 2014) to minimize the impact of induced prism effects in users’ vision. To our best knowledge, no previous report has described the assessment (repeatability and agreement) of any device that clinically measures the eye’s visual axis. This study is the first to assess the repeatability and agreement using the current gold standard (modified Viktorin’s method (Walsh & Pearce, 2009) using a traditional frame ruler) of a new prototype (Ergofocus®; Lentitech, Barakaldo, Spain) designed to clinically measure the eye’s visual axis (namely, FFA).

Previous reports have described the repeatability of different methods (standard and modified Viktorin’s method (Walsh & Pearce, 2009; Holland & Siderov, 1999)) and devices (standard ruler, pupillometer, and other devices (McMahon, Irving & Lee, 2012; Garcia-Espinilla et al., 2022; Holland & Siderov, 1999; Wesemann, 2010)) to measure the nasopupillary distance in ophthalmic lens practice. These reports (McMahon, Irving & Lee, 2012; Holland & Siderov, 1999) have described interpupillary distance repeatability (Sw) between 0.56 and 0.69 mm measured using a frame ruler, which is slightly lower than the Sw achieved using FFA measurement. However, the frame ruler has a limited precision to 1.0 mm, and the Ergofocus device has a 0.10 mm measuring step according to the manufacturer’s information. Other devices have been proposed for nasopupillary distance and/or interpupillary distance measurement in clinical practice, such as the pupillometer (PD-2 pupillometer (BON), digital CRP pupillometer (Essilor, Charenton-le-Pont, France), Pm-100 pupillometer (Rodenstock, Munich, Germany), and PD-5 pupilometer (Topcon, Shinjuku City, Japan) (Garcia-Espinilla et al., 2022; Wesemann, 2010), image apps (Opticenter (Prats Optical, Sant Boi de Llobregat, Spain)), Visureal portable (Ollendorf) and others) (Garcia-Espinilla et al., 2022; Wesemann, 2010) or specific devices (such as Visioffice (Essilor, Charenton-le-Pont, France), ImpressionIST (Rodenstock, Munich, Germany), Visureal (Hoya, Ollendorf, Germany), RVT (Zeiss), and others) (Garcia-Espinilla et al., 2022; Wesemann, 2010). These devices have shown repeatability (Sw) coefficients between approximately 0.10 and 0.50 mm. Therefore, the repeatability of FFA measurements is acceptable clinically (with a CV lower than 5% and high ICC). However, new versions of this prototype could improve the repeatability of its measurements.

The agreement of FFA measurement with the nasopupillary distance measured using a traditional frame ruler is low with higher differences in near vision than in far vision. This difference is expected because different measurement approaches are used by each technique, the frame ruler uses the pupil centre, the new prototype uses the visual axis, and the difference between both distances forms the kappa angle (Chang & Waring, 2014; Rabbetts, 2007). Previous reports have described angle kappa distances between 0.3 and 0.9 mm (Basmak et al., 2007a; Domínguez-Vicent et al., 2014; Meng et al., 2021; Pande & Hillman, 1993; Bonaque-González et al., 2021) (distance between the corneal light reflex and the pupil centre); thus, the pupil centre usually does not match the eye visual fixation axes. Because ophthalmic lenses are usually placed 12 mm from the corneal apex, this difference will likely be higher than the angle kappa distance measured at the corneal apex plane. A simple ray tracing approximation, assuming a mean anterior chamber depth of 3.0 mm (Leng et al., 2014), allows the assumption that angle kappa distances between 0.3 and 0.9 mm could represent distances between 1.5 and 4.5 mm in the ophthalmic lens plane. Therefore, differences between traditional frame ruler and FFA measurements could not be attributable just to the new prototype and angle kappa could be one of the main reasons for the differences obtained. Additionally, slight differences in kappa angle with strabismus (Basmak et al., 2007b), refractive error (Yeo, Moon & Lee, 2017) and age (Yeo, Moon & Lee, 2017) have been described; consequently, the difference between the nasopupillary distance and FFA will be larger as the kappa angle increases.

This low agreement (Fig. 4) seems to confirm that FFA and nasopupillary distance are not interchangeable measurements because the FFA should coincide with visual axis and links the fixation point and fovea (regardless of the area of the pupil) directly. However, nasopupillary distance considers the pupillary centre as reference.

### Clinical implications

Differences between nasopupillary distance and FFA values could be relevant in different clinical scenarios. For example, undesired prismatic effects would occur that increase in high refractive errors (Flores, 2009) because, according to ISO 21897:2017, the maximum accepted horizontal prismatic effect error caused by a centration error is 0.67 prismatic dioptres. Therefore, according to Prentice’s law (Tang, 1989), the increased distance to the optical centre of the lens and increase in the dioptric power lens imply an increase in the prismatic effect. Additionally, because the mean difference found in this study in the right eye was higher than 2.0 mm at far distances, only a prescription of 3.50 D could fail with the recommended tolerance described in ISO 21897:2017. Furthermore, the difference was higher in near vision (close to 3.0 mm in both right and left eyes) and a minimum decentering of a lens of just 2.25 D could induce a prismatic effect that was higher than that tolerated by ISO.

In summary, the mean difference between the nasopupillary distance and FFA found in this study would suggest a breach of ISO 21897:2017 rule with moderate and high prescriptions. Therefore, more studies assessing FFA, nasopupillary distance and kappa angle are necessary to assess the impact on spectacle wearers.

A second major scenario could be presbyopia management with PAL because these lens prescriptions require correct and accurate facial measurements to minimize subjects’ adaptation process because of the reduced intermediate and near vision zones in these lenses explained by the Minkwitz theorem (Sheedy et al., 2005; Esser et al., 2017). Therefore, accurately centering the PAL with the patient’s visual axis at far and near distances to avoid aberration zones in the visual field is critical (Han, Graham & Lin, 2011) to reduce the subject’s PAL inadaptation rate and drop-out (Odjimogho & Odjimogho, 2011). The clinical use of FFA measurements could allow better centering of the PAL and could decrease the PAL inadaptation rate, achieving better vision performance. Further research assessing the subject’s PAL adaptation rate using FFA measurements is necessary.

### Study limitations

The main study limitation could be the sample size comprising Caucasian subjects aged between 40 and 65 years. However, this sample could be adequate to conduct the repeatability and agreement analysis in this study following previous recommendations (Carkeet, 2015), showing 95% confidence intervals for the repeatability Bland–Altman LoA (Carkeet, 2015) and including regression analysis to assess the effect of the overall magnitude on the differences between devices (agreement) or repeated measurements (repeatability).

Additionally, the absence of other published clinical methods to measure the visual axis made it challenging to compare these study results. Only one recent report (Kim et al., 2018) proposed a new method to determine the visual axis *in vivo* based on dual-depth whole-eye optical coherence tomography that is unavailable for clinical practice and does not measure distances between visual axes to prescribe ophthalmic lenses. However, the main interest of previous reports was assessing the repeatability of nasopupillary distance and/or interpupillary distance measurements collected using different methods or devices that use different measurement principles (mainly pupil centre) that could not be compared with the Ergofocus prototype that uses the FFA for measurements.

## Conclusion

FFA measurements showed clinically acceptable repeatability at both far and near distances with significant differences from the nasopupillary distance and interpupillary distance measured using a standard frame ruler, suggesting that both measurements are not interchangeable in clinical practice to prescribe and centre ophthalmic lenses. Further research to assess the use of FFA outcomes in clinical practice is required with special attention in PAL prescription to explore whether FFA measurements could improve user satisfaction, minimize the subject PAL adaptation process, and replace current nasopupillary distance and interpupillary distance use to centre this type of lens.

## Supplemental Information

10.7717/peerj.14942/supp-1 Supplemental Information 1 Dataset for agreement analysis.

10.7717/peerj.14942/supp-2 Supplemental Information 2 Dataset for repeatability analysis.

## Additional Information and Declarations

### Data Availability

The following information was supplied regarding data availability: The raw data, measurements used for repeatability and agreement analysis, are available in the Supplemental Files.
